# Comparing expedient and proactive approaches to the planning of protected area networks on Borneo

**DOI:** 10.1038/s44185-024-00052-8

**Published:** 2024-08-21

**Authors:** Ewan A. Macdonald, Samuel A. Cushman, Yadvinder Malhi, David W. Macdonald

**Affiliations:** 1 Worcester College, University of Oxford, Walton St, Oxford, OX1 2HB UK; 2 Saïd Business School, University of Oxford, Park End Street, Oxford, OX1 1HP UK; 3 Wildlife Conservation Research Unit, Biology Department, University of Oxford, The Recanati-Kaplan Centre, Tubney House, Abingdon Road, Tubney, Abingdon, OX13 5QL UK; 4 Environmental Change Institute, School of Geography and the Environment, University of Oxford, South Parks Road, Oxford, OX1 3QY UK

**Keywords:** Conservation biology, Ecological modelling

## Abstract

Protected areas are an important tool for wildlife conservation; however, research is increasingly revealing both biases and inadequacies in the global protected area network. One common criticism is that protected areas are frequently located in remote, high-elevation regions, which may face fewer threats compared to more accessible locations. To explore the conservation implications of this issue, we consider a thought experiment with seven different counterfactual scenarios for the Sunda clouded leopard’s conservation on Borneo. This allows us to examine two contrasting paradigms for conservation: “proactive conservation” which prioritises areas with high biodiversity and high risk of development, and “expedient conservation” which focusses on areas with the lowest development risk. We select clouded leopards as our focal species not only because of their emerging conservation importance, but also because, as top predators, they represent both keystone species and ambassadors for wider forest biodiversity. Furthermore, a published analysis of the likely impacts of forest loss in their habitat provides a benchmark for evaluating the modelled outcomes of alternative hypothetical conservation scenarios. We find that, across all metrics, expedient reserve design offered few benefits over the business-as-usual scenario, in contrast to the much greater conservation effectiveness of proactive protected area design. This paper sheds light on the challenging trade-offs between conservation goals and the competing land uses essential for the economic development and well-being of local communities.

## Introduction

Protected areas play a crucial role in wildlife conservation around the world. Recognising this does not diminish the value of habitats beyond protected area boundaries or the effectiveness of land-sharing approaches aimed at promoting harmonious human-wildlife coexistence^1,2^; nevertheless, the identification and protection of special areas for conservation remain a cornerstone of conservation policy^3^. Despite these efforts, many protected areas may not fully fulfil their intended goals: for instance, Lindsey et al. ^4^. estimated that most lion populations remaining in protected areas existed at scarcely half their carrying capacity. Additionally, an extensive and growing literature discusses so-called “paper parks” that provide little to no actual protection^5^, along with increasing human pressures within protected areas^6^. Understanding the factors that influence protected area effectiveness is thus widely seen as a conservation priority^6^.

One important question is how to maximise conservation benefits via the optimal location and design of protected areas. Early work on this question focused on concepts from the Theory of Island Biogeography. Through the 1990s and early 2000s, an intense debate occurred about whether it was better to protect a few large areas or many small areas (e.g. Diamond Vs Simberloff & Abele ^7^ summarised in Tjørve^8^). This debate was never resolved, largely because it was rarely addressed with rigorous analytical, modelling or empirical data. The recent “consensus” on the topic is well encapsulated by Lawton’s maxim “*more, bigger, better and joined*”^9^. However, this maxim might be recited more in hope than expectation, given the real and severe socio-political, economic and geographical constraints to designating vast networks of large and well-connected protected areas.

Spatial conservation prioritisation^10^ is a valuable tool for designing reserves to explore and balance the trade-offs. In the context of a rapidly increasing human footprint and economic growth, policy-makers should consider detailed, specific, and quantitative evaluations of alternative conservation design strategies. These strategies should optimize the location of new protected areas for maximum biodiversity benefit^11^, while also delivering wider goals such as the preservation of ecosystem processes and functions^12,13^, providing ecosystem services such as the preservation of forest carbon stocks^14^ and alleviating poverty^15^. These approaches have been used in a variety of contexts in Borneo, including attempts to evaluate the effectiveness of Sarawak’s protected area network^16^, develop reserve designs that balance economic development with biodiversity conservation^17^, and understand the vulnerability of the Bornean protected area network to climate change^18,19^.

Historically, the placement of protected areas has usually been driven more by expediency than by their contribution to conservation goals^20^. This has often resulted in a protected area infrastructure that is biased towards locations that are often inaccessible, of limited economic value, and functionally poor, often referred to as “high and far” or “rocks and ice”^13^. As a result, these areas are often unlikely to face development pressures, regardless of their formal protection status^21,22^. Joppa and Pfaff^21^ summarize the challenge: if protected areas are to change land-use outcomes, they need to be in places most at risk from land conversion, the very opposite of high and far! This is especially true considering that many of the most biodiverse regions of the planet are experiencing some of the highest human pressures^23^ and are under the greatest threat^24^. Planning protected areas involves many factors, including biology, economics, politics, development, law, international relations, and ethics. It is important to acknowledge all these aspects as we look at two logical extremes in the enduring debate about reserve design: expediency and proactivity.

We define proactive conservation as placing new protected areas in regions with high immediate risk of forest loss, while expedient conservation focuses on areas with low immediate risk. We hypothesise that although proactive approaches may encounter greater economic and political challenges, they are likely to produce more significant conservation benefits amidst rapid land use change. To our knowledge, no previous studies have sought to quantify the impact of these two paradigmatic approaches to reserve design. Here, we use a variety of hypothetical scenarios to quantitatively compare the potential impact of these two approaches on Sunda clouded leopards and forest carbon stocks in Borneo.

We chose this example species and system due to the valuable insights gained from over a decade of research to identify priority areas for the conservation of clouded leopards across the range of the two species (*Neofelis nebulosa* and *Neofelis diardi*) (e.g. Macdonald, et al. ^25^. and Macdonald, et al. ^26^) and forecasting the consequences of alternative development policies^27–30^. Clouded leopards are a suitable focal species for this assessment not only because their conservation is an emerging concern^31^, but also because they serve as a model for top predators which deliver important ecosystem services^32^ and may act as potent ambassadors for wider biodiversity in the region^33,34^.

Several studies have attempted to predict future forest loss on the island of Borneo^35,36^ however, these typically use a logistic regression based approach that fails to utilize an explicitly multi-scale optimization (sensu McGarigal, et al. ^37^). Cushman et al. ^38^ used a multi-scale random forest machine learning model to predict risk of forest loss in 2020 for Borneo based on observed patterns of forest loss from 2000 and 2010, considering topography, land cover, human population density, and conservation status. They found that this approach consistently outperformed logistic regression, and that including landscape structure variables substantially improved predictions^38^. The resulting forest loss risk map indicated the likelihood that a forested pixel in 2010 would be lost by 2020. We used those predictions as a ‘business-as-usual’ benchmark to assess the probable consequences of forest loss on factors such as the population size and genetic variability of Sunda clouded leopards, as well as the remaining stock of forest carbon under each of our scenarios. This provided a robust foundation for posing a series of thought experiments about theoretical, yet relevant, conservation strategies. We chose these dates to exemplify the principles as they form a well-defined time series with available data. However, the main purpose of this paper is to explore the logical extremes of a well-known but seldom quantified trade-off in conservation. Therefore, the specific dates in this example are not material to the core conclusions.

Our methodology combines resistant kernel and spatially synoptic least cost path approaches (UNICOR^39,40^) to identify the most important core areas and corridors for the Sunda clouded leopard across Borneo. We then use individual-based population and genetic simulation (CDPOP^41^) to compare the relative importance of these areas in maintaining clouded leopard population size and genetic diversity.

The scenarios we examine address key questions related to land use policy. Firstly, can we quantify and compare the relative impact of proactive and expedient approaches to conservation, in this case using the example of the Sunda clouded leopard? Secondly can we assess the effectiveness of the existing protected area network for clouded leopards?

We examined various counterfactual scenarios for the conservation of clouded leopards (detailed in Table 1). Scenario 1 represented the baseline condition. Scenarios 2 and 3 represented a ‘proactive’ approach to conservation situating new protected areas in areas of imminent high risk. In contrast, scenarios 4 and 5 represented an ‘expedient’ approach, placing new protected areas in regions with the lowest risk of forest loss and thus presumably have the lowest cost of conservation. Within these proactive and expedient scenarios, we also considered the impact of the existing protected area network on the reserve design. Scenarios 2 and 4 redistributed the existing protected areas and ‘started from scratch’, while scenarios 3 and 5 were forced to retain the existing protected area network in the reserve design. Finally, scenarios 6 and 7 retained the existing protected area network but assumed that no new protected areas would be added. Of these, scenario 6 assumed that the existing protected area network would be effective and that these areas will be invulnerable to forest loss. In contrast, scenario 7 assumed that these protected areas would be exposed to the risks of forest loss predicted by Cushman et al. ^38^, which accounts for protection status as one of the predictor variables.

**Table 1:** Description of scenarios described in this analysis

No	Scenario	Description	Map
1	Baseline	This scenario represents the status of clouded leopard habitat (forest – green) in 2010.	
2	Proactive conservation redistributing PAs	This scenario identifies a total of 17% of Borneo’s land area as new protected areas (yellow), designed from scratch to deliver optimal benefits for the conservation of both clouded leopards and forest carbon in areas at highest risk of forest loss. All PAs in this scenario are treated as being 100% effective at preventing forest loss within their boundaries. Forest loss proceeds as predicted in unprotected forest areas.	
3	Proactive conservation maintaining PAs	This scenario expands upon Borneo’s existing PA network (red) to protect a total of 17% of Borneo’s land area, new PAs (yellow) are designed to deliver optimal benefits for the conservation of both clouded leopards and forest carbon in areas at highest risk of forest loss. All PAs in this scenario are treated as being 100% effective at preventing forest loss within their boundaries. Forest loss proceeds as predicted in unprotected forest areas.	
4	Expedient conservation redistributing PAs	This scenario identifies a total of 17% of Borneo’s land area as new protected areas (blue), designed from scratch to deliver optimal benefits for the conservation of both clouded leopards and forest carbon in areas at lowest risk of forest loss. All PAs in this scenario are treated as being 100% effective at preventing forest loss within their boundaries. Forest loss proceeds as predicted in unprotected forest areas.	
5	Expedient conservation maintaining PAs	This scenario expands upon Borneo’s existing PA network (red) to protect a total of 17% of Borneo’s land area, new PAs (blue) are designed to deliver optimal benefits for the conservation of both clouded leopards and forest carbon in areas at lowest risk of forest loss. All PAs in this scenario are treated as being 100% effective at preventing forest loss within their boundaries. Forest loss proceeds as predicted in unprotected forest areas.	
6	Existing PAs effective	This scenario relies entirely on Borneo’s existing network of PAs (red) but assumes that all PAs in this scenario are 100% effective at preventing forest loss within their boundaries. Forest loss proceeds as predicted in unprotected forest areas.	
7	Business-as-usual	This scenario relies entirely on Borneo’s existing network of PAs (semi-transparent red) but assumes that PAs will experience forest loss in line with that predicted by the forest loss risk model. Forest loss will also proceed as predicted in unprotected forest areas.	

Green areas show planning units classified as forest, grey areas are classified as not forest, and red areas show the location of existing protected areas. Yellow (proactive) and blue (expedient) areas indicate the location of new protected areas as identified by the MARXAN optimization.

Under the expedient scenarios (4 and 5), we asked “which areas provide optimal conservation benefits for both clouded leopards and forest carbon at the lowest risk?”. We anticipated that these scenarios would propose conservation areas in central Borneo but that high-risk lowland areas would likely be lost to other land uses. In the proactive conservation scenarios (2 and 3), we asked, “which areas deliver optimal conservation benefits for both clouded leopards and forest carbon stocks while minimising the probable loss of forests?” We expected the proactive scenarios to suggest conservation actions in the most vulnerable areas of Sabah and Sarawak. The central Bornean highlands are not likely to be a priority under this scenario. However, these areas are also classified as low risk (predominantly because they are high in elevation, topographically rough, and far from existing deforested areas) and are therefore unlikely to be lost within the timescale of this analysis.

The full details of the data layers and modelling approach are provided in the methods section. Briefly, we used MARXAN^42^, a widely used decision support tool, to evaluate seven different scenarios aimed at optimising the conservation outcomes for both clouded leopards on Borneo, and forest carbon conservation. Then UNICOR^40^, a connectivity modelling tool, and CDPOP^41^, a spatially explicit individual-based population and genetic model, were used to quantify changes in habitat connectivity and assess the impacts of these changes on projected clouded leopard population size and genetic diversity.

## Results

### Validation of forest loss risk model

Validation of the Cushman et al. ^38^. forest loss risk layer against the Hansen et al. ^43^ map of observed forest loss resulted in an AUC (Area Under the Curve) metric of 0.71. This is equivalent to the proportion of pixels classified correctly and indicates high overall accuracy. Sensitivity, representing the proportion of correctly classified forest loss pixels was found to be lower than specificity, which denotes the proportion of accurately classified no-change pixels (sensitivity = 0.51, specificity = 0.76). This suggests a greater ease in predicting areas where forest loss did not occur compared to those where it did. Overall, the validation affirmed a high level of accuracy in our forest loss risk model. The error rates observed are consistent with the stochastic nature of forest loss (i.e. we can be confident that a cell with a low probability of loss will remain forested, however, when two cells each have a 50% probability of loss it is difficult to tell which one will be lost). Full validation results, including changes in overall rates and the spatial distribution of forest loss are presented in the supplementary material.

### Carbon

**Fig. 1:**
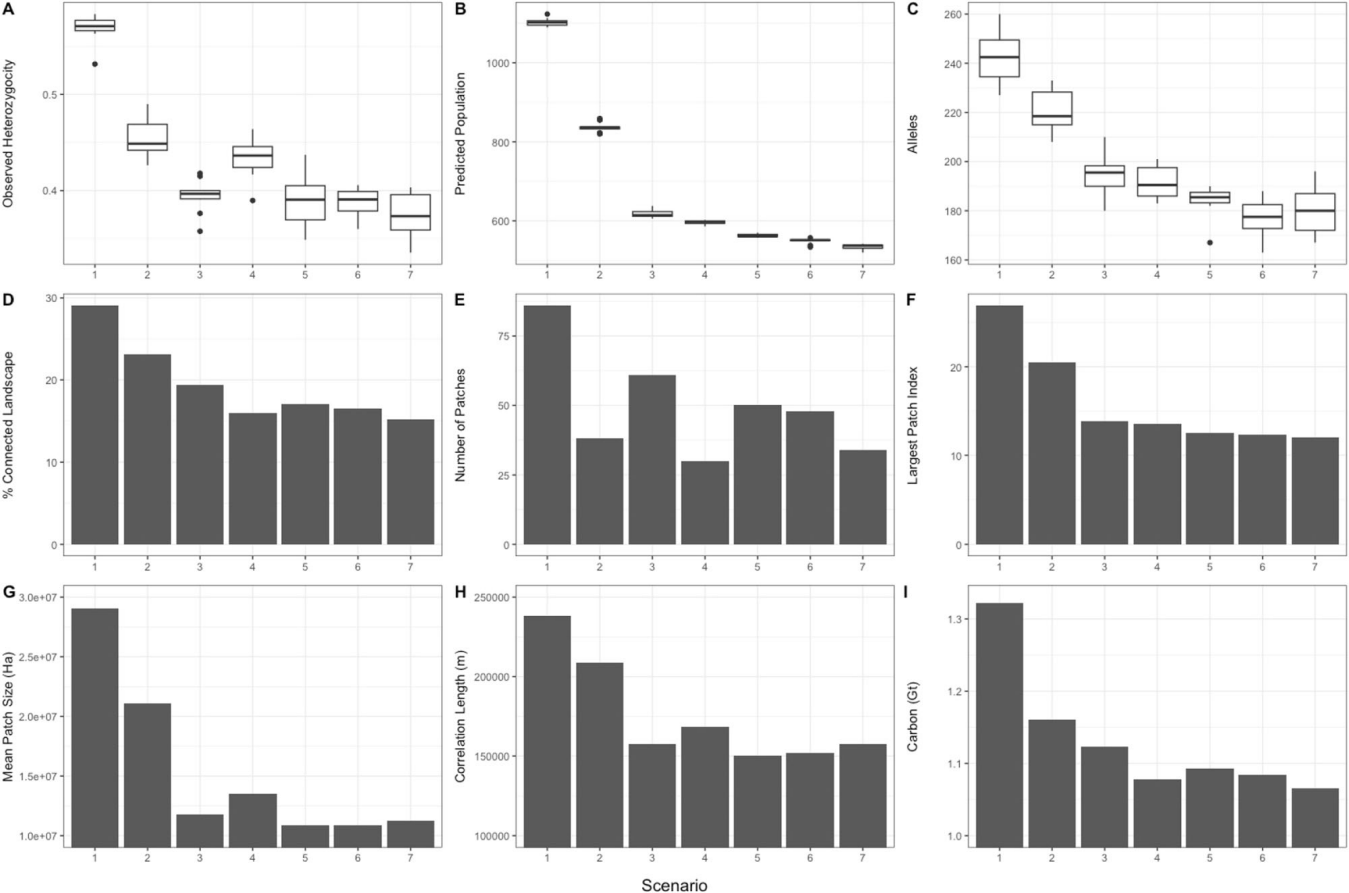
Comparison of genetic and landscape metrics, and forest carbon stocks for each of the different scenarios. The various panels show (**A**) boxplot of observed heterozygosity, (**B**) boxplot of predicted population, (**C**) boxplot of number of alleles, (**D**) percentage of connected landscape, (**E**) number of patches, (**F**) largest patch index, (**G**) mean patch size, (**H**) correlation length, (**I**) carbon. Scenarios listed on the x-axis correspond to Table 1 and Fig. 2. The central bar on the boxplots corresponds to the median value, upper and lower hinges correspond to the 1st and 3rd quartiles, whiskers extend from the hinge to the value no further than 1.5 * inter quartile range from the hinge^73^.

The impact of different scenarios on forest carbon stocks reveals a substantial reduction in carbon storage across all scenarios, decreasing from approximately 1.32 gigatons (Gt) in the baseline to between 1.07 and 1.15 Gt in the six alternative scenarios (Fig. 1I). While still experiencing large losses in forest carbon, the proactive scenarios (S2 and S3) performed best, while scenarios 4 and 7 (expedient conservation redistributing protected areas (PAs), and business-as-usual) exhibited the lowest efficacy in maintaining forest carbon stocks.

### Resistant Kernel connectivity

**Fig. 2:**
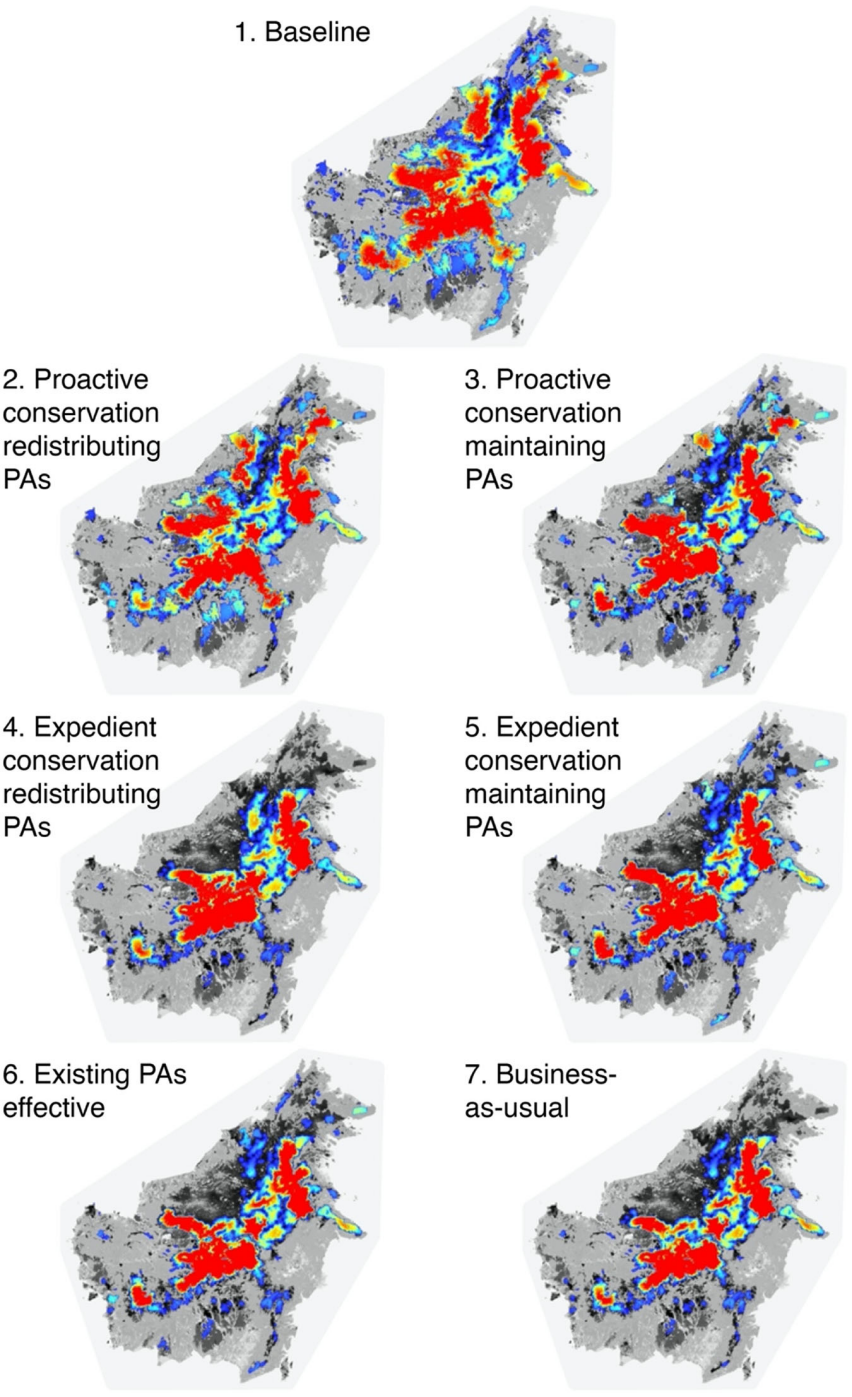
Modelled Sunda clouded leopard kernel densities under a number of different scenarios. (1) Baseline, (2) Proactive conservation redistributing PAs, (3) Proactive conservation maintaining PAs, (4) Expedient conservation redistributing PAs, (5) Expedient conservation maintaining PAs, (6) Existing PAs effective, (7) Business-as-usual. Maps show relative colour ramp from low (blue) to high (red) and grey scale background is predicted not to be core area in our UNICOR model. Scenarios correspond to Table 1.

The resistant kernel connectivity analysis indicated substantial changes in the expected density and distribution of clouded leopard dispersal movements across the seven scenarios (Fig. 2).

Across all measures, the baseline scenario (S1) had the highest indices of connectivity, with the remaining scenarios exhibiting varying levels of decline. The percentage of the landscape connected by dispersal was 29% in the baseline but fell to 15.2% under the business-as-usual scenario (S7). Comparing proactive and expedient approaches reveals that both proactive scenarios retain a greater proportion of connected landscape than the expedient scenarios (proactive: S2: 23.2%, S3: 19.4% Vs expedient S4: 16.0%, S5: 17.0%). Indeed, the expedient scenarios offer very few gains compared to business as usual. The largest patch index (the extent of largest patch of connected habitat as a percentage of the landscape) was 26.9% in the baseline scenario (S7) but fell to 20.5% in the next best performing scenario: proactive conservation redistributing PAs (S2; Fig. 1F). All other scenarios experienced similar, large, declines with scores below 14%. Area weighted mean patch size of connected habitat was highest in the baseline scenario (S7: 29.0 million ha) and experienced the smallest decline in the proactive scenario that redistributed PAs (S2: 21.1 million ha; Fig. 1G); all other scenarios decline to between 10.8 and 13.5 million ha. The correlation length of connected habitat was greatest in the baseline scenario (238.5 km) and declined least in the proactive scenario that redistributed PAs (S2: 208.9 km; Fig. 1H) all other scenarios decline to between 150.4 to 168.6 km. The number of patches of isolated habitat was highest in the baseline scenario (S7: 86) and declines in all other scenarios (Fig. 1E). The lowest number of isolated habitat patches occurred in the expedient scenario that redistributed PAs (S4: 30) due to the high concentration and compactness in that scenario. Scenarios that protected the existing protected area network (S3, S5 and S6) all had a higher number of patches due to the fragmented nature of the existing reserve design.

### CDPOP Simulations

The CDPOP simulations of changes in population size and genetic diversity across the seven scenarios also reveal substantial differences among the alternative conservation strategies. After 200 generations at the low dispersal ability (125,000 cost units), our simulation projected a total population size of about 1100 clouded leopards under the baseline scenario (S7; Fig. 1B). In all other scenarios, this population estimate was projected to be substantially smaller due to anticipated landscape changes. The proactive conservation scenario that redistributed PAs had the second highest predicted population (S2: 840 individuals) while all other scenarios exhibited markedly worse performance with projected populations sizes in the range of 650-530 individuals. This pattern of results was mirrored at the high dispersal ability (250,000 cost units; Supplementary Fig. 3).

As expected, allelic richness was again highest in the baseline scenario (S1: 242.5) and declined across all others. Allelic richness was 9.1% lower in the proactive scenario that redistributed PAs making it second ranking scenario (S2: 220.4; Fig. 1C). Scenarios 5, 6, and 7 (Expedient conservation maintaining PAs, Existing PAs effective, and Business-as-usual) all had similar levels of allelic richness (177.2–183.9), suggesting that an expedient approach to increasing the existing network of protected areas would do little to increase overall allelic richness. There was no clear relationship between dispersal ability and allelic richness, with the total number of alleles in the population being similar between the low (125,000 cost unit) and high (250,000 cost unit) dispersal thresholds (Supplementary Fig. 2).

All scenarios are projected to result in large reductions in observed heterozygosity compared to the baseline (S1: 0.57; Fig. 1A). The two scenarios that allowed for the redistribution of protected areas (S2: Proactive conservation redistributing PAs, and S4: Expedient conservation redistributing PAs) resulted in the smallest loss in observed heterozygosity (S2: 0.45, S4: 0.43). The two scenarios that retained the existing protected area network both had lower, and similar, levels of heterozygosity (S3: 0.39, S5: 0.39). This suggests that incorporating existing protected areas results in lower overall population-wide heterozygosity because it protects a more dispersed and fragmented network of protected areas each of which experiences a greater loss of genetic diversity due to isolation effects and genetic drift. In all cases, the larger dispersal ability (250,000 cost units) resulted in higher predicted heterozygosity than the lower dispersal ability (125,000 cost units; Supplementary Fig. 1).

## Discussion

This study uses the specific case of Sunda clouded leopards in Borneo to illustrate general principles about the design of protected area networks. Our analysis quantitatively compares the impact of proactive and expedient approaches to conservation, illustrating how expedient conservation strategies are much less effective at conserving connectivity, population size, and genetic diversity than proactive conservation designs. Furthermore, we show how the existing protected area network in Borneo appears to be highly suboptimal for the conservation of the apex carnivore, the clouded leopard.

The results show that the business-as-usual scenario would result in rapidly deteriorating conditions for Sunda clouded leopards. Specifically, this scenario projected a landscape capable of supporting approximately half the current clouded leopard population. Declines were also predicted for two measures of genetic diversity along with a projected ~20% loss of forest carbon stocks equating to a loss of 256.12million tons of carbon stored as above ground biomass.

It is important to note that these projections are derived from a simulation model run for 200 generations to provide a standardized, objective comparative yardstick. This does not suggest that implementing a proactive conservation strategy would conserve 50% more clouded leopards over short, decadal time scales relevant to policy and conservation planning. Rather, it shows that, on a relative basis, expedient conservation strategies result in landscapes that are 50% less effective in supporting the population than proactive measures.

In summary, our analysis predicts that the business-as-usual scenario will lead to a widespread decline in clouded leopard numbers, likely compounded by dramatic declines in genetic diversity.

The business-as-usual scenario presented in this analysis acknowledges the presence of Borneo’s protected area network but assumes that these areas will experience forest loss at rates similar to those predicted within protected areas by Cushman et al. ^38^. If we assume that these protected areas were effective at preventing forest loss, as depicted in Scenario 6 (Existing PAs effective), we predict a 9% increase in the extent of the connected landscape relative to the business-as-usual scenario (Figure 2.7). However, under scenario 6, clouded leopards would still experience widespread population declines and decreases in genetic diversity (Fig. 1A–C). This highlights that, although the existing protected area network is currently insufficient to provide adequate protection of clouded leopards, effectively protecting these areas against illegal logging and encroachment would still offer small but measurable conservation benefits.

Comparing the remaining scenarios reveals that Scenario 2, which involves proactive conservation through redistributing PAs, is predicted to provide major conservation benefits for clouded leopards. This scenario is projected to deliver a 53% improvement in the percentage of the landscape connected by dispersal relative to the business-as-usual scenario. It would substantially reduce habitat fragmentation by creating fewer, larger, discreet patches of habitat than many other scenarios. Scenario 2 also exhibited the best performance in terms of the simulated population size and genetic diversity, resulting in a landscape that would likely support 57% more clouded leopards and 9% more forest carbon compared to the business-as-usual scenario.

While we acknowledge that Scenario 2 (proactive conservation redistributing PAs) represents an unrealistic option for policymakers, it nonetheless carries an important message. Of all the scenarios, this is the only one that substantially improved outcomes for clouded leopard conservation relative to the business-as-usual scenario. All the other scenarios predict worsening conditions, though there is variation between them. Thus, while it seems unlikely that either proactive or expedient conservation designs can reverse the negative trend for clouded leopard habitat and populations, this shows the importance of approaches that lessen the slope and severity of habitat loss.

Comparing proactive and expedient approaches to conservation (scenarios 2 vs 4, and 3 vs 5) reveals that, across all metrics, proactive conservation outperforms expedient conservation design. For instance, considering the scenarios that allow for the reallocation of protected areas (scenarios 2 vs 4), the proactive approach results in a landscape that is 53% more connected than the business-as-usual scenario, while the expedient scenario only results in a landscape that is 5% more connected. The proactive scenario redistributing PAs (scenario 2) is predicted to result in improved population size and genetic diversity for clouded leopards, and the conservation of an extra 82 million tonnes of CO_2_ of forest carbon compared to the expedient scenario that redistributes PAs.

While these scenarios represent currently unrealistic logical extremes of reserve design, they nevertheless illustrate a broader point. Considering the more realistic scenarios that add to the existing protected area network (Scenarios 3 vs 5), we still observe important improvements with proactive conservation. The proactive scenario results in a landscape that is 28% more connected than the business-as-usual scenario, while the expedient scenario results in a landscape that is only 12% more connected than the business-as-usual scenario. Additionally, the proactive scenario led to significant improvements in population size and genetic diversity for clouded leopard populations, as well as the conservation of an extra 29 million tonnes of forest carbon.

Here, we wish to emphasize our point to avoid being misunderstood. We dismiss neither the beauty nor biodiversity value of the existing protected area network, nor do we seek to belittle or undermine the motives of those who strove to gazette them. However, we point out that the vast majority of protected lands in Borneo, and possibly worldwide, are located in remote and rugged highland areas that are currently unassailable by economically viable anthropogenic land-use change (though they might become threatened in future). While we do not undervalue these protected areas, we emphasize that, over the timescale considered, their formal protection adds little in terms of habitat protection (distinct from, for example, anti-poaching efforts). Securing them does not represent a significant victory for conservation over competitive land uses. This is why we term this strategy expediency.

We use the clouded leopard dataset as an exemplar, recognising the species’ pivotal role as a top-predator and its reflection of other elements of biodiversity^33^. However, we do not suggest redesigning the entire reserve network to suit one species, even if it is a potent ambassador^34^. Nonetheless, if a more assertive strategy of proactive conservation were imagined for Borneo, designed to answer the question of what (re)deployment of resources, would yield the greatest gain for clouded leopards and the forest biodiversity associated with them, the resulting map would look very different.

The wider issues explored here have global relevance. Whether conservationists aspire to meet the goals of the Kunming-Montreal Global Biodiversity Framework or to go further and reserve “half earth” for biodiversity^44^, understanding the factors that contribute to protected area effectiveness remains a critical priority for global conservation efforts^6^. The logical extremes presented here are relevant to conservation planners and policy makers considering issues ranging from the preservation of the world’s last wilderness areas^45^ to the development of more effective and more representative marine reserves (e.g. Jantke, et al. ^46^).

Understanding the implications of potential counterfactual conservation scenarios, such as modelling the impact of locating protected areas in regions of high or low risk of forest loss, offers insight for conservation prioritisation^21,47^. Focusing protection on more threatened areas could intensify already difficult trade-offs between the goals of conservation and those of social justice, along with the provision of goods and services important for the wellbeing of local communities^48^. Conservationists cannot expect biodiversity gain to be a trump card in land-use policy, but the debate might be fruitfully realigned by considering the impact of different counterfactual scenarios, rather than settling for the line of least resistance.

This begs an obvious question: since expedient approaches to protected area design offer little additional benefit to conservation, how can we make proactive approaches more attractive and relevant to policy makers? One simple and obvious way to improve outcomes would be to enhance protected area effectiveness and reduce unauthorized and illegal land conversion within these areas. However, our results show that this would only produce a relatively small improvement compared to the range of scenarios modelled.

Furthermore, in many parts of the world, land use change occurs because the net returns on extractive, human-dominated uses are higher than the current economic or social returns from biodiversity^49^. In such cases, the opportunity costs of conservation are likely to be far higher under proactive approaches^50^. In the immediate term, several policy options exist to narrow this gap, including payments for ecosystem services schemes such as REDD+. A recent review of opportunity costs for REDD+ concluded that these initiatives can often be cost-effective but the opportunity costs are highly variable and driven by a variety of forces^51^. Our results also highlight the importance of additionality in such schemes. Additionally, the capacity for REDD+ projects to deliver biodiversity benefits on top of habitat conservation varies across species and threats^52^.

Despite these promising avenues, it thus seems that there will still be places that warrant proactive conservation and where additional incentives alone will fail bridge the cost gap. Often, this gap represents a form of market failure where the developer does not bear the true social and environmental costs of land conversion. Therefore, policy interventions aimed at internalising these externalities, such as the polluter pays principle, perhaps through targeted taxation or the elimination of perverse subsidies, are critical to reducing the relative cost of proactive conservation approaches^53^. Balancing the costs of conservation is challenging and requires novel approaches to environmental economic thinking^54–56^.

## Methods

### Clouded leopard core areas

Macdonald et al. ^31^ used a resistant kernel approach^39^ to identify potential core areas for the Sunda clouded leopard across Borneo for the years 2000, 2010 and 2020. The choice of these specific years was guided by data availability and facilitated a clear time series analysis. However, it is important to note that the primary aim of this study is to examine the theoretical implications of different conservation paradigms, rendering the specific dates less pivotal to the core conclusions. The resistant kernel approach, which predicts the relative density of dispersing individuals in each pixel across the study area, incorporates factors such as species dispersal ability, the nature of the dispersal function, and landscape resistance^57^. Areas exhibiting high cumulative resistant kernel scores are interpreted as potential core areas for clouded leopards or corridors for dispersal, whereas those with lower scores are less likely to be utilized by the species. Because little is known about clouded leopard dispersal, Macdonald, et al. ^31^ calculated resistant kernel values for two dispersal distances representing plausible upper (250 km) and lower (125 km) bounds for maximum dispersal distance.

In this analysis, we utilized the cumulative resistant kernel map for the year 2010 as provided by Macdonald et al. ^31^, with a dispersal distance of 125 km. The choice of the 125 km dispersal distance was based on it’s alignment with empirically derived home range data for clouded leopards on Borneo^58,59^, which served as a reliable basis for estimating dispersal distances. This distance was considered the most suitable fit for our study’s objectives and the characteristics of the study area.

### Risk of forest loss

Estimates of the risk of forest loss were obtained from Cushman et al. ^38^. This analysis employed a multi-scale random forest machine learning approach^60^ to predict risk of forest loss in 2020, based on observed patterns of forest loss in relation to topographical features, landcover, human population density, and conservation status of land between 2000 and 2010. This forest loss risk map provided the probability that a pixel, that was forested in 2010 would be lost by 2020.

The risk of forest loss was validated against observed forest loss during the same period by comparing Cushman et al. ^38^ to Hansen et al. ^43^ to assess model performance^61,62^. Specifically, the validation treated the forest loss data from Hansen, et al. ^43^ between 2010 and 2020 as “truth”. Pixels showing forest loss in Hansen et al. ^43^ between 2010 and 2020 were coded as 1, while forest pixels that were not lost during this period were coded as 0. We extracted the predicted forest loss risk from Cushman et al. ^38^ and the observed forest loss (0/1) from Hansen et al. ^43^ at 1,000,000 randomly chosen locations across Borneo.

Using the PresenceAbsence package^63,64^ we computed several performance metrics, including the area under the Receiver Operating Characteristic Curve (AUC), sensitivity, specificity, and percentage of correctly classified observations at the cut-point that maximized the Kappa statistic. The AUC measures the model’s ability to distinguish between forest loss and no loss, sensitivity indicates the true positive rate, and specificity represents the true negative rate.

### Forest carbon stocks

Estimates of forest carbon stocks were obtained from Saatchi, et al. ^65^ who provide a benchmark map of forest carbon stocks in the tropics. This dataset includes estimates for above-ground biomass, below-ground biomass, and total biomass carbon at a 1 km resolution. For our analysis, we focused on Above Ground Biomass (AGB) since many carbon finance schemes exclude below-ground biomass from their calculations. This exclusion is often due to the challenges in accurately measuring below-ground biomass and its lesser relevance in carbon trading mechanisms.

### Protected areas

A map of protected areas was obtained from the World Database on Protected Areas^66^. Our analysis included only those protected areas classified under IUCN categories I-VI, which range from strict nature reserves (category I) to protected areas with sustainable use of natural resources (category VI). We included only those areas with associated shape files to ensure spatial accuracy and consistency in our analysis.

### Forest vs not forested areas

Given that clouded leopards are closely associated with forest habitats^25^, we excluded predominantly non-forest areas from our MARXAN analysis, as they were not considered suitable habitat. We identified forest and not forest areas based on data from Miettinen et al. ^67^. Areas classified as forest areas included mangrove, peat swamp forest, lowland forest, lower montane forest, and upper montane forest. Conversely, areas classified as not forest comprised water, plantation/regrowth, lowland mosaic, mountain mosaic, lowland open, montane open, urban, and large-scale palm plantations. These habitat types are considered to be suboptimal for clouded leopards^31^ and were thus classed as not forest.

All data layers were resampled to a resolution of 500 m and scaled from 0 to 1 by dividing by their maximum value. MARXAN analyses were conducted using a 10 × 10 km grid of planning units across the island of Borneo. In total there were 7675 planning units. The vast majority (89.8%) covered an area of 100 km^2^, while the remaining 10.2% were located in coastal areas and thus covered a smaller land area.

**Fig. 3:**
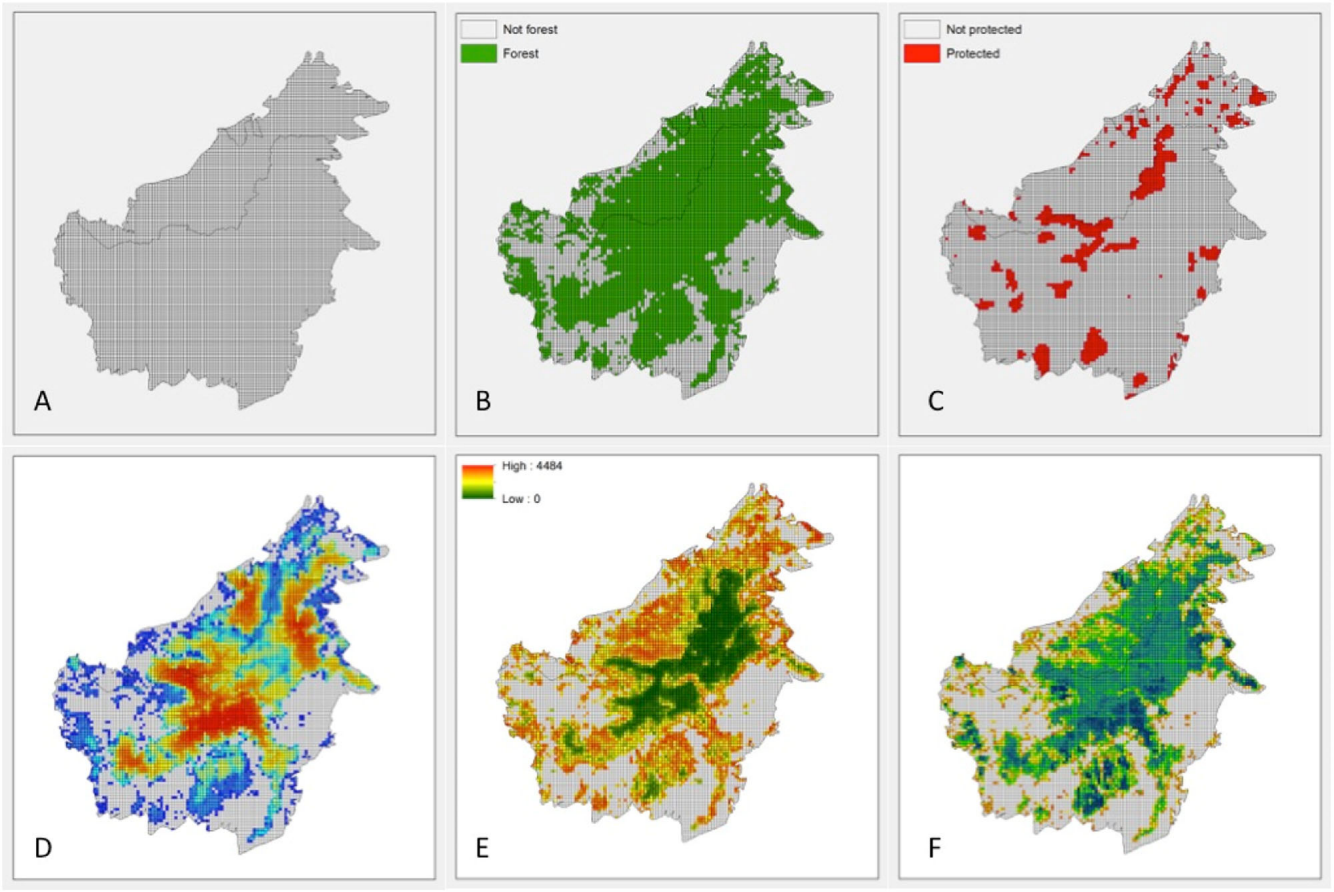
Data layers used in analysis. **A** Map of planning units across Borneo, (**B**) planning units classified as Forest^38^, (**C**) planning units classified as protected^66^, (**D**) sum kernel value in each planning unit^31^, (**E**) mean risk of forest loss in each planning unit^38^, (**F**) Sum carbon value (above ground biomass) in each planning unit^65^.

For each planning unit, we calculated the area classified as forest, the area covered by PAs, the sum of the resistant kernel score, the sum of above ground biomass, and the mean risk of forest loss (Fig. 3).

Although little is known about the spatial ecology of clouded leopards, one radio-collared female was estimated to have a home range of 16.1 km^2^ ^59^. Therefore, any planning units that did not contain at least 16 km^2^ of forested habitat were excluded from the reserve design solutions. Similarly, only planning units that containing more than 16 km^2^ of existing protected areas were considered to be protected.

### Scenarios

We considered several contrasting scenarios for clouded leopard conservation. Scenario 1 represented the baseline condition. Scenarios 2 and 3 represented a ‘proactive’ approach, placing new protected areas in regions of imminent high risk. In contrast, Scenarios 4 and 5 represented an ‘expedient’ approach, locating new protected areas in regions with the lowest risk of forest loss, presumably reducing the cost of conservation.

Within these ‘proactive’ and ‘expedient’ approaches, we also considered the impact of the existing protected area network on the reserve design. Scenarios 3 and 5 retained the existing protected area network and identified new priority areas for expansion. Conversely, Scenarios 2 and 4 reimagined the protected area network by redistributing the existing PAs across an entirely new design, essentially starting from scratch.

Finally, scenarios 6 and 7 retained the existing protected area network but assumed no new protected areas would be added. Scenario 6 assumed that the existing PA network would be effective and invulnerable to forest loss, while Scenario 7 assumed these PAs would be subject to the forest loss risks predicted by Cushman et al. ^38^, which includes protection status as a predictor variable.

Under the expedient scenarios (S4 and S5), we asked, “Which areas provide optimal conservation benefits for both clouded leopards and carbon at the lowest risk?” We expected these scenarios to suggest conservation actions in central Borneo predicting that high risk lowland areas would be lost to other land uses. Under the proactive conservation scenarios (S2 and S3), we asked, “Which areas provide optimal conservation benefits for both clouded leopards and forest carbon stocks while minimising likely forest losses?” We anticipated that these scenarios would suggest conservation actions in the most vulnerable areas of Sabah and Sarawak. The central Bornean highlands are unlikely to be prioritised under this approach because they are classified as low risk (due to their high in elevation, rough topography, and distance from existing deforested areas) and are therefore unlikely to be lost within analysis timescale.

### MARXAN parameterisation

We used the decision support tool MARXAN^42^, running in Zonae Cogito^68^, to identify the optimal placement of new protected areas for scenarios 2, 3, 4 and 5. While clouded leopards might occasionally traverse human-dominated land uses^27^, they depend on forest for habitat^69^. Therefore, all forested planning units were available for selection, while all ‘not forest’ planning units were excluded from the reserve design. For scenarios that retained the existing protected area network (scenarios 3 and 5), planning units containing more than 16 km^2^ of protected areas were locked into the reserve design.

Clouded leopard resistant kernel density and forest carbon stocks were included as ‘conservation features’. In the proactive conservation scenarios (scenarios 2 and 3), the risk of forest loss was also included as a ‘conservation feature’, and the ‘cost penalty’ for each planning unit was proportional to it’s area (assuming equal costs of conservation across all areas). In the expedient conservation scenarios (scenarios 4 and 5), the ‘cost penalty’ was set to be proportional to the mean risk of forest loss in each unit (assuming higher opportunity costs for high-risk planning units).

To ensure comparability between scenarios, we used the same MARXAN parameters throughout. These values were identified through several calibration runs and provided the most realistic conservation scenarios^70^.

- **Conservation Targets**: The target for each conservation feature, representing the minimum amount that must be included within the reserve network to meet conservation objectives, was set to 30%. This ensured an equal weighting between features.
- **Species Protection Factor (SPF)**: The SPF, which represents the penalty imposed by MARXAN if a scenario fails to meet the conservation target for a species, was set to 2. This setting ensured that all targets were met in over 99% of model runs.
- **Boundary Length Modifier (BLM)**: The BLM is a weighting that adjusts the ‘clumpiness’ of the solution. It was set to 0.0015, which was sufficient to yield a realistic level of clumping in the reserve design, avoiding both a ‘salt and pepper’ reserve design and a single clumped reserve.

Each MARXAN scenario involved 1,000,000 iterations and 10,000 runs.

In each run MARXAN identified a different configuration of planning units, and across all runs, the frequency with which planning units were selected was taken as a measure of conservation priority. Since our goal was to protect 17% of Borneo’s land area (or 1305 of the total 7657 planning units), our final reserve design included the 1305 planning units with the highest selection frequency for that scenario. In scenarios 2 and 3, MARXAN was forced to include the 944 planning units containing existing reserves and could allocate an additional 311 units to reach our area target of 17%. In scenarios 4 and 5, MARXAN was free to allocate all 1305 units to the optimal reserve design.

### Comparison of scenario Solutions

Following Macdonald et al. ^31^, we used the forest loss risk map to project changes in landscape resistance between different points in time and applied the resistant kernel model to infer the effect of these changes on clouded leopard core areas. This was achieved by multiplying the probability of forest loss for each cell by the resistance value for the “plantations and regrowth” land use class (creating the expected mean increase in resistance) and then adding this value to the existing resistance value in the baseline condition. Forest pixels that were lost were most likely to transition to plantations or regrowth. All areas included within our reserve networks were considered to be effectively protected (excluding scenario 7) and retained the same resistance values as in the baseline condition, while all other areas were adjusted to account for their risk of forest loss. This conservatively assumes that there was no leakage, meaning the protection of forest in one area did not result in the commensurate loss of forest in another.

Following Macdonald et al. ^31^, we used UNICOR^40^ to calculate the cumulative resistant kernel density for each resistance map at 125,000 cost units (125kcu), and at 250,000 cost units (250kcu). The resistant kernel approach to connectivity modelling is based on all-directional least-cost dispersal from a defined set of source points^39^. The resistance maps for the three dates provide resistances for all locations in the study area, indicating the increased cost of crossing each pixel relative to the least-cost condition. These costs are used as weights in the dispersal function, meaning the expected density of dispersing individuals in a pixel is down-weighted by the cumulative cost from the source, following the least-cost route^39^. The initial expected density was set to 1 in each cell containing a source point. The model calculates the expected relative density of dispersers in each pixel around the source, given the dispersal ability of the species, the nature of the dispersal function, and the resistance of the landscape^39,71^.

In their raw form, the resistant kernel density maps depict the expected density of dispersing individuals, indicating the distribution of connected populations. A threshold kernel density is necessary to delineate connected populations. We used a threshold value of the 10^th^ percentile to reflect clouded leopard core areas (all areas above the 10^th^ percentile in resistant kernel density) and reclassified the six kernel density maps into a binary form, assigning a value of 1 where the kernel density probability of dispersal is greater than each percentile threshold and 0 where it is less.

We used FRAGSTATS^72^ to calculate the percentage of the landscape, number of patches, largest patch index, area weighted mean patch size, and correlation length of the landscape connected by dispersal for clouded leopards. The percentage of the landscape quantifies how much of the study area is predicted to be connected habitat for each kernel map. The largest patch index^72^ reports the extent, as a proportion of the study area, of the largest patch of connected habitat. The area weighted mean patch size^72^ calculates the average size of each patch of connected habitat, with large patches weighted more heavily than small patches to reflect greater ecological importance. Lastly, correlation length is the average distance an organism can move within a patch from a random starting point before encountering the edge of the patch.

Following Macdonald et al. ^31^ we used CDPOP 1.0^41^ to simulate clouded leopard mating and dispersal as functions of the spatial patterns of resistance across the seven scenarios. We extracted several global measures of population genetic structure for the full study area after 200 generations of CDPOP simulation time. The point of the simulation is not to estimate clouded leopard populations 200 generations into the future but to compare quantitatively the effects of different habitat conservation scenarios involving different degrees of protection. These measures included observed heterozygosity, total number of alleles in the population, and population size. We analyzed the differences in these global measures of population size and genetic structure between the scenarios. It is important to note that the simulation is not intended to predict the actual changes in genetic diversity of population size over a short decadal time period of the immediate future, but to provide a comparable, biologically-based yardstick to contrast the impacts of alternative scenarios in terms of their performance in population and genetic conservation.

Finally, we considered the impact of all seven scenarios on the above-ground biomass carbon stocks in Borneo’s forests (i.e., in all areas classified as forest). For each of the scenarios, we calculated the carbon values in each cell by reducing the current (2010) carbon values by the product of current carbon and the inverse probability of that cell being deforested. In scenario 7, we assumed that existing protected areas were subject to the level of risk predicted by the risk surface (which includes protected area status as a predictor variable^38^). In all other scenarios, protected areas were assumed to be effective and retained their 2010 carbon values.

### Supplementary information

Supplementary information

## Data Availability

The datasets used and/or analysed during the current study are available from the corresponding author on reasonable request.

## References

[1] Tyrrell, P., du Toit, J. T. & Macdonald, D. W. Conservation beyond protected areas: Using vertebrate species ranges and biodiversity importance scores to inform policy for an east African country in transition. *Conserv. Sci. Pract.* **2**, e136 (2020). 10.1111/csp2.136

[2] Western, D. et al. Conservation from the inside-out: Winning space and a place for wildlife in working landscapes. *People Nat.* **2**, 279–291, 10.1002/pan3.10077 (2020). 10.1002/pan3.10077

[3] Watson, J. E. M., Dudley, N., Segan, D. B. & Hockings, M. The performance and potential of protected areas. *Nature* **515**, 67–73, 10.1038/nature13947 (2014). 25373676 10.1038/nature13947

[4] Lindsey, P. A. et al. The performance of African protected areas for lions and their prey. *Biol. Conserv.* **209**, 137–149, 10.1016/j.biocon.2017.01.011 (2017). 10.1016/j.biocon.2017.01.011

[5] Di Minin, E. & Toivonen, T. Global protected area expansion: Creating more than paper parks. *BioScience* **65**, 637–638, 10.1093/biosci/biv064 (2015). 26955080 10.1093/biosci/biv064 PMC4776719

[6] Jones, K. R. et al. One-third of global protected land is under intense human pressure. *Science* **360**, 788–791, 10.1126/science.aap9565 (2018). 29773750 10.1126/science.aap9565

[7] Diamond, J. M. The island dilemma: Lessons of modern biogeographic studies for the design of natural reserves. *Biol. Conserv.* **7**, 129–146, 10.1016/0006-3207(75)90052-X (1975). 10.1016/0006-3207(75)90052-X

[8] Tjørve, E. How to resolve the SLOSS debate: Lessons from species-diversity models. *J. Theor. Biol.* **264**, 604–612, 10.1016/j.jtbi.2010.02.009 (2010). 20152842 10.1016/j.jtbi.2010.02.009

[9] DEFRA. Making Space for Nature: a review of England’s wildlife sites and ecological network. (2010).

[10] Moilanen, A., Wilson, K. & Possingham, H. (Oxford University Press, Oxford; New York, 2009).

[11] Ferrier, S. & Wintle, B. In *Spatial Conservation Prioritization Quantitative Methods and Computational Tools* (eds Atte Moilanen, Kerrie Wilson, & Hugh Possingham) (Oxford University Press, 2009).

[12] Bennett, A. F. et al. Ecological processes: A key element in strategies for nature conservation. *Ecol. Manag. Restor.* **10**, 192–199, 10.1111/j.1442-8903.2009.00489.x (2009). 10.1111/j.1442-8903.2009.00489.x

[13] Fynn, R. W. S. & Bonyongo, M. C. Functional conservation areas and the future of Africa’s wildlife. *Afr. J. Ecol.* **49**, 175–188, 10.1111/j.1365-2028.2010.01245.x (2011). 10.1111/j.1365-2028.2010.01245.x

[14] De Barros, A. E. et al. Identification of areas in Brazil that Optimize Conservation of Forest Carbon, Jaguars, and Biodiversity. *Conserv. Biol.* **28**, 580–593, 10.1111/cobi.12202 (2014). 24372997 10.1111/cobi.12202

[15] Suich, H., Howe, C. & Mace, G. Ecosystem services and poverty alleviation: A review of the empirical links. *Ecosyst. Serv.* **12**, 137–147, 10.1016/j.ecoser.2015.02.005 (2015). 10.1016/j.ecoser.2015.02.005

[16] Lawes, M. J. et al. In *RIMBA3: Sustaining livelihoods through prudent utilization and management of natural resources* (eds Tuen A. A., Mohd-Azlan J., & Grinang J.) (Institute of Biodiversity and Environmental Conservation, Universiti Malaysia Sarawak, 2014).

[17] Runting, R. K. et al. Alternative futures for Borneo show the value of integrating economic and conservation targets across borders. *Nat. Commun.* **6**, 6819, 10.1038/ncomms7819 (2015). 25871635 10.1038/ncomms7819 PMC4403346

[18] Scriven, S. A., Hodgson, J. A., McClean, C. J. & Hill, J. K. Protected areas in Borneo may fail to conserve tropical forest biodiversity under climate change. *Biol. Conserv.* **184**, 414–423, 10.1016/j.biocon.2015.02.018 (2015). 10.1016/j.biocon.2015.02.018

[19] Struebig, Matthew J. et al. Targeted conservation to safeguard a biodiversity hotspot from climate and land-cover change. *Curr. Biol.* **25**, 372–378, 10.1016/j.cub.2014.11.067 (2015). 25619764 10.1016/j.cub.2014.11.067

[20] Ando, A., Camm, J., Polasky, S. & Solow, A. Species distributions, land values, and efficient conservation. *Science* **279**, 2126–2128, 10.1126/science.279.5359.2126 (1998). 9516117 10.1126/science.279.5359.2126

[21] Joppa, L. N. & Pfaff, A. High and far: Biases in the location of protected areas. *PLOS ONE* **4**, e8273, 10.1371/journal.pone.0008273 (2009). 20011603 10.1371/journal.pone.0008273 PMC2788247

[22] Venter, O. et al. Bias in protected-area location and its effects on long-term aspirations of biodiversity conventions. *Conserv. Biol.* **32**, 127–134, 10.1111/cobi.12970 (2018). 28639356 10.1111/cobi.12970

[23] Venter, O. et al. Sixteen years of change in the global terrestrial human footprint and implications for biodiversity conservation. *Nat. Commun.* **7**, 12558, 10.1038/ncomms12558 (2016). 27552116 10.1038/ncomms12558 PMC4996975

[24] Newbold, T. et al. Global effects of land use on local terrestrial biodiversity. *Nature* **520**, 45–50, 10.1038/nature14324 (2015). 25832402 10.1038/nature14324

[25] Macdonald, D. W. et al. Multi-scale habitat selection modeling identifies threats and conservation opportunities for the Sunda clouded leopard (Neofelis diardi). *Biol. Conserv.* **227**, 92–103, 10.1016/j.biocon.2018.08.027 (2018). 10.1016/j.biocon.2018.08.027

[26] Macdonald, D. W. et al. Multi-scale habitat modelling identifies spatial conservation priorities for mainland clouded leopards (Neofelis nebulosa). *Diversity Distrib.* **25**, 1639–1654, 10.1111/ddi.12967 (2019). 10.1111/ddi.12967

[27] Hearn, A. J. et al. Evaluating scenarios of landscape change for Sunda clouded leopard connectivity in a human dominated landscape. *Biol. Conserv.* **222**, 232–240, 10.1016/j.biocon.2018.04.016 (2018). 10.1016/j.biocon.2018.04.016

[28] Kaszta et al. Integrating Sunda clouded leopard (Neofelis diardi) conservation into development and restoration planning in Sabah (Borneo). *Biol. Conserv.* **235**, 63–76, 10.1016/j.biocon.2019.04.001 (2019). 10.1016/j.biocon.2019.04.001

[29] Kaszta et al. Simulating the impact of Belt and Road initiative and other major developments in Myanmar on an ambassador felid, the clouded leopard, Neofelis nebulosa. *Landsc. Ecol.* **35**, 727–746, 10.1007/s10980-020-00976-z (2020). 10.1007/s10980-020-00976-z

[30] Kaszta, Ż., Cushman, S. A. & Macdonald, D. W. Prioritizing habitat core areas and corridors for a large carnivore across its range. *Anim. Conserv.* **23**, 607–616, 10.1111/acv.12575 (2020). 10.1111/acv.12575

[31] Macdonald, E. A. et al. Simulating impacts of rapid forest loss on population size, connectivity and genetic diversity of Sunda clouded leopards (Neofelis diardi) in Borneo. *PLOS ONE* **13**, e0196974, 10.1371/journal.pone.0196974 (2018). 30208031 10.1371/journal.pone.0196974 PMC6135353

[32] Estes, J. A., Tinker, M. T., Williams, T. M. & Doak, D. F. Killer whale predation on sea otters linking oceanic and nearshore ecosystems. *Science* **282**, 473–476, 10.1126/science.282.5388.473 (1998). 9774274 10.1126/science.282.5388.473

[33] Chiaverini, L. et al. Multi-scale, multivariate community models improve designation of biodiversity hotspots in the Sunda Islands. *Anim. Conserv.* **25**, 660–679, 10.1111/acv.12771 (2022). 10.1111/acv.12771

[34] Macdonald, E. A. et al. Identifying ambassador species for conservation marketing. *Glob. Ecol. Conserv.* **12**, 204–214, 10.1016/j.gecco.2017.11.006 (2017). 10.1016/j.gecco.2017.11.006

[35] Struebig, M. J. et al. Anticipated climate and land-cover changes reveal refuge areas for Borneo’s orang-utans. *Glob. Change Biol.* **21**, 2891–2904, 10.1111/gcb.12814 (2015). 10.1111/gcb.12814 25559092

[36] Voigt, M. et al. Deforestation projections imply range-wide population decline for critically endangered Bornean orangutan. *Perspect. Ecol. Conserv.* **20**, 240–248, 10.1016/j.pecon.2022.06.001 (2022). 10.1016/j.pecon.2022.06.001

[37] McGarigal, K., Wan, H. Y., Zeller, K. A., Timm, B. C. & Cushman, S. A. Multi-scale habitat selection modeling: A review and outlook. *Landsc. Ecol.* **31**, 1161–1175, 10.1007/s10980-016-0374-x (2016). 10.1007/s10980-016-0374-x

[38] Cushman, S. A., Macdonald, E. A., Landguth, E. L., Malhi, Y. & Macdonald, D. W. Multiple-scale prediction of forest loss risk across Borneo. *Landsc. Ecol.* **32**, 1581–1598, 10.1007/s10980-017-0520-0 (2017). 10.1007/s10980-017-0520-0

[39] Compton, B. W., Mcgarigal, K., Cushman, S. A. & Gamble, L. R. A resistant-Kernel model of connectivity for amphibians that breed in vernal pools. *Conserv. Biol.* **21**, 788–799, 10.1111/j.1523-1739.2007.00674.x (2007). 17531056 10.1111/j.1523-1739.2007.00674.x

[40] Landguth, E. L., Hand, B. K., Glassy, J., Cushman, S. A. & Sawaya, M. A. UNICOR: a species connectivity and corridor network simulator. *Ecography* **35**, 9–14, 10.1111/j.1600-0587.2011.07149.x (2012). 10.1111/j.1600-0587.2011.07149.x

[41] Landguth, E. L. & Cushman, S. A. cdpop: A spatially explicit cost distance population genetics program. *Mol. Ecol. Resour.* **10**, 156–161, 10.1111/j.1755-0998.2009.02719.x (2010). 21565001 10.1111/j.1755-0998.2009.02719.x

[42] Ball, I. R., Possingham, H. P. & Watts, M. In *Spatial Conservation Prioritization: Quantitative Methods and Computational Tools* (eds Atte Moilanen, Kerrie Wilson, & Hugh Possingham) 185-195 (Oxford University Press, 2009).

[43] Hansen, M. C. et al. High-resolution global maps of 21st-century forest cover change. *Science* **342**, 850–853, 10.1126/science.1244693 (2013). 24233722 10.1126/science.1244693

[44] Wilson, E. O. *Half-earth : our planet’s fight for life*. First edition edn, 259 pages : illustrations ; 25 cm (Liveright Publishing Corporation, a division of W.W. Norton & Company New York, 2016).

[45] Allan, J. R., Venter, O. & Watson, J. E. M. Temporally inter-comparable maps of terrestrial wilderness and the Last of the Wild. *Sci. Data* **4**, 170187, 10.1038/sdata.2017.187 (2017). 29231923 10.1038/sdata.2017.187 PMC5726312

[46] Jantke, K. et al. Poor ecological representation by an expensive reserve system: Evaluating 35 years of marine protected area expansion. *Conserv. Lett.* **11**, e12584, 10.1111/conl.12584 (2018). 10.1111/conl.12584

[47] Arlidge, W. N. S. et al. A global mitigation hierarchy for nature conservation. *BioScience* **68**, 336–347, 10.1093/biosci/biy029 (2018). 29731513 10.1093/biosci/biy029 PMC5925785

[48] Vucetich, J. A. et al. Just conservation: What is it and should we pursue it? *Biol. Conserv.* **221**, 23–33, 10.1016/j.biocon.2018.02.022 (2018). 10.1016/j.biocon.2018.02.022

[49] Du Toit, J. T., Cross, P. C. & Valeix, M. In *Rangeland Systems: Processes, Management and Challenges* (ed David D. Briske) 395-425 (Springer International Publishing, 2017).

[50] Tyrrell, P., Naidoo, R., Macdonald, D. W. & du Toit, J. T. New forces influencing savanna conservation: increasing land prices driven by gentrification and speculation at the landscape scale. *Front. Ecol. Environ.* **19**, 494–500, 10.1002/fee.2391 (2021). 10.1002/fee.2391

[51] Yang, H. & Li, X. Potential variation in opportunity cost estimates for REDD+ and its causes. *For. Policy Econ.* **95**, 138–146, 10.1016/j.forpol.2018.07.015 (2018). 10.1016/j.forpol.2018.07.015

[52] Collins, M. B., Milner-Gulland, E. J., Macdonald, E. A. & Macdonald, D. W. Pleiotropy and Charisma Determine Winners and Losers in the REDD+ Game: All Biodiversity is Not Equal. *Tropical Conserv. Sci.* **4**, 261–266, 10.1177/194008291100400304 (2011). 10.1177/194008291100400304

[53] Helm, D. Natural capital: assets, systems, and policies. *Oxf. Rev. Econ. Policy* **35**, 1–13, 10.1093/oxrep/gry027 (2019). 10.1093/oxrep/gry027

[54] Bateman, I. J. & Mace, G. M. The natural capital framework for sustainably efficient and equitable decision making. *Nat. Sustainability* **3**, 776–783, 10.1038/s41893-020-0552-3 (2020). 10.1038/s41893-020-0552-3

[55] Raworth, K. *Doughnut economics : seven ways to think like a 21st century economist*. 309 pages : illustrations ; 23 cm (Chelsea Green Publishing White River Junction, Vermont, 2017).

[56] Vucetich, J. A. et al. A minimally nonanthropocentric economics: What is it, is it necessary, and can it avert the biodiversity crisis? *BioScience* **71**, 861–873, 10.1093/biosci/biab045 (2021). 10.1093/biosci/biab045

[57] Cushman, S. A., Shirk, A. & Landguth, E. L. Separating the effects of habitat area, fragmentation and matrix resistance on genetic differentiation in complex landscapes. *Landsc. Ecol.* **27**, 369–380, 10.1007/s10980-011-9693-0 (2012). 10.1007/s10980-011-9693-0

[58] Bowman, J., Jaeger, J. A. G. & Fahrig, L. Dispersal distance of mammals is proportional to home range size. *Ecology* **83**, 2049–2055, 10.1890/0012-9658(2002)083[2049:DDOMIP]2.0.CO;2 (2002). 10.1890/0012-9658(2002)083[2049:DDOMIP]2.0.CO;2

[59] Hearn, A. et al. Insights into the spatial and temporal ecology of the Sunda clouded leopard Neofelis diardi. *Raffles Bull. Zool.* **61**, 871–875 (2013).

[60] Evans, J. S. & Cushman, S. A. Gradient modeling of conifer species using random forests. *Landsc. Ecol.* **24**, 673–683, 10.1007/s10980-009-9341-0 (2009). 10.1007/s10980-009-9341-0

[61] Pontius Jr, R. G. & Si, K. The total operating characteristic to measure diagnostic ability for multiple thresholds. *Int. J. Geogr. Inf. Sci.* **28**, 570–583, 10.1080/13658816.2013.862623 (2014). 10.1080/13658816.2013.862623

[62] Pontius, R. G. & Parmentier, B. Recommendations for using the relative operating characteristic (ROC). *Landsc. Ecol.* **29**, 367–382, 10.1007/s10980-013-9984-8 (2014). 10.1007/s10980-013-9984-8

[63] Freeman, E. A. & Moisen, G. PresenceAbsence: An R package for presence absence analysis. *J. Stat. Softw.* **23**, 1–31, 10.18637/jss.v023.i11 (2008). 10.18637/jss.v023.i11

[64] R: A Language and Environment for Statistical Computing (R Foundation for Statistical Computing, Vienna, Austria, 2021).

[65] Saatchi, S. S. et al. Benchmark map of forest carbon stocks in tropical regions across three continents. *Proc. Natl. Acad. Sci.* **108**, 9899–9904, 10.1073/pnas.1019576108 (2011). 21628575 10.1073/pnas.1019576108 PMC3116381

[66] IUCN & UNEP-WCMC. The World Database on Protected Areas (WDPA). (2014).

[67] Miettinen, J., Shi, C., Tan, W. J. & Liew, S. C. 2010 land cover map of insular Southeast Asia in 250-m spatial resolution. *Remote Sens. Lett.* **3**, 11–20, 10.1080/01431161.2010.526971 (2012). 10.1080/01431161.2010.526971

[68] Segan, D. B., Game, E. T., Watts, M. E., Stewart, R. R. & Possingham, H. P. An interoperable decision support tool for conservation planning. *Environ. Model. Softw.* **26**, 1434–1441, 10.1016/j.envsoft.2011.08.002 (2011). 10.1016/j.envsoft.2011.08.002

[69] Hearn, A. J. et al. Responses of Sunda clouded leopard Neofelis diardi population density to anthropogenic disturbance: refining estimates of its conservation status in Sabah. *Oryx* **53**, 643–653, 10.1017/S0030605317001065 (2019). 10.1017/S0030605317001065

[70] Ardron, J. A., Possingham, H. P. & Klein, C. J. (Pacific Marine Analysis and Research Association, Victoria, BC, Canada, 2010).

[71] Cushman, S. A., Chase, M. & Griffin, C. In *Spatial Complexity*, *Informatics, and Wildlife Conservation* (eds Samuel A. Cushman & Falk Huettmann) 349-367 (Springer Japan, 2010).

[72] FRAGSTATS v4: Spatial Pattern Analysis Program for Categorical and Continuous Maps. (University of Massachusetts, Amherst, 2012).

[73] Wickham, H. ggplot2: Elegant Graphics for Data Analysis. (2016).

