# Supplementary material for: Comparing expedient and proactive approaches to the planning of protected area networks on Borneo
Source: NPJ Biodivers. 2024 Aug 21;3:20. doi: 10.1038/s44185-024-00052-8 (PMC11339377; doi:10.1038/s44185-024-00052-8)
Supplement: Supplementary file 1 — Supplementary information [file 44185_2024_52_MOESM1_ESM.pdf]

Supplementary material:

Comparison of results at two dispersal abilities:

The figures contained in this section compare the results of analyses at two dispersal abilities: 125,000 and 250,000 cost units.

Seven Scenarios (see Table 1 in the main manuscript for a full description):

1: Baseline
2. Proactive conservation redistributing PAs
3: Proactive conservation maintaining PAs
4: Expedient conservation redistributing PAs
5: Expedient conservation maintaining PAs
6: Existing PAs effective
7: Business-as-usual

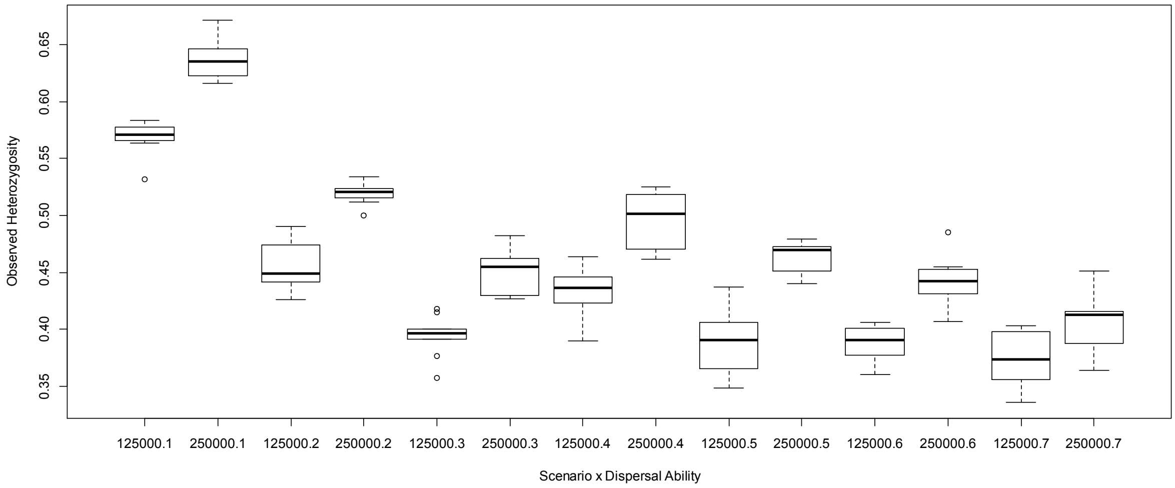

Supplementary Figure 1. Boxplots showing differences in observed heterozygosity in the simulated population after 200 generations for each scenario across dispersal abilities. All scenarios are predicted to result in large reductions in observed heterozygosity. All scenarios that designated additional protected areas improved heterozygosity relative to the alternatives that did not (scenarios 6 and 7). The Proactive conservation scenario that redistributes PAs (scenario 2) and the expedient conservation scenario that redistributes PAs (scenario 4) were the scenarios that resulted in the smallest loss in observed heterozygosity. The two scenarios that retained the existing protected area network both had lower and similar, levels of heterozygosity. This indicates that retaining existing protected areas results in lower overall population-wide heterozygosity because it protects a more dispersed and fragmented network of protected areas which each experience a higher loss of genetic diversity due to isolation effects and genetic drift. In all cases, the larger

dispersal ability (250,000 cost units) resulted in higher predicted heterozygosity than the lower dispersal ability (125,000 cost units).

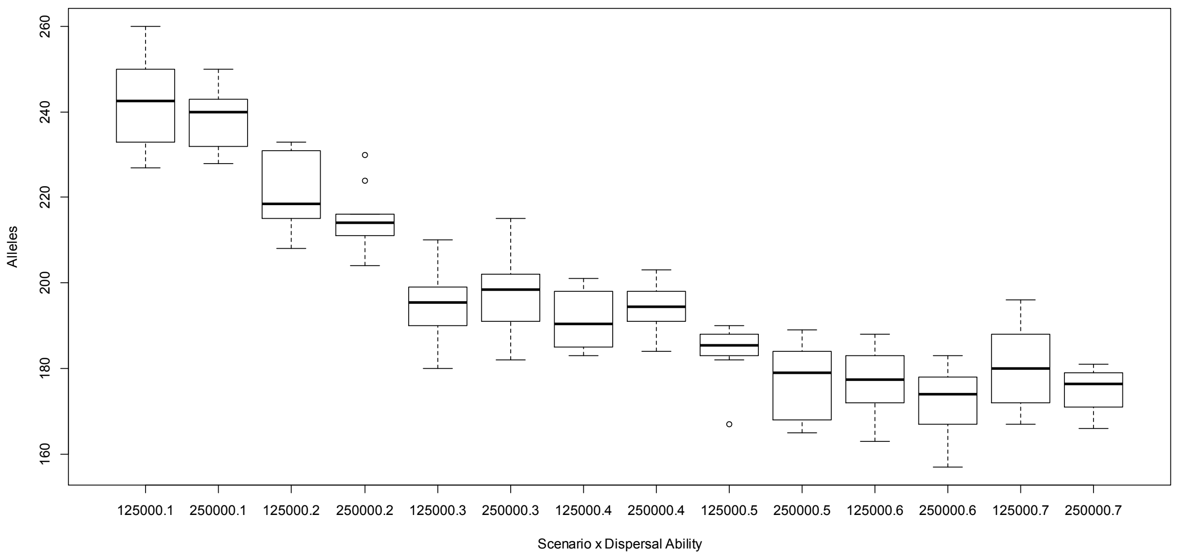

Supplementary Figure 2. Boxplots showing differences in the number of alleles in the in simulated population after 200 generations for each scenario across dispersal abilities. The proactive conservation scenario that redistributes PAs (scenario 2) resulted in substantially higher allelic richness than any other scenario, amounting to a reduction of approximately 10% from the 2010 scenario. Scenarios 3 and 4 (proactive conservation maintaining PAs, and expedient conservation redistributing PAs) had roughly equivalent simulated allelic richness (approximately 18% reduction from the baseline scenario). Scenarios 5, 6, and 7 had similar reductions of allelic richness, indicating the that expedient scenario that retained existing protected areas did not result in improved total allelic diversity in the population over the scenarios that did not protect any additional protected areas. There was no clear relationship between dispersal ability and allelic richness, with the total number of alleles in the population similar between the low (125,000 cost unit) and high (250,000 cost unit) scenarios for each protected area conservation strategy.

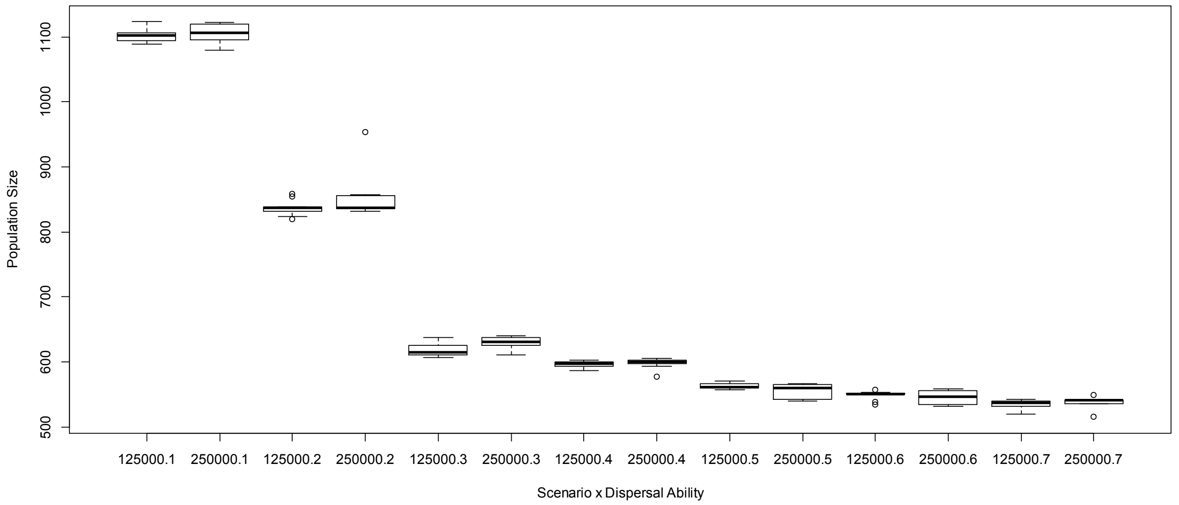

Supplementary Figure 3. Boxplots showing differences in the total population size of the simulated population after 200 generations for each scenario across dispersal abilities. After 200 generations our simulation projected a total population size of about 1100 clouded leopards at both the high and low dispersal abilities under the baseline landscape condition. The population size was predicted to decrease dramatically under all scenarios due to predicted landscape change. Scenario 2 had by far the least predicted population decline as compared to the other scenarios. Specifically, both the high and low dispersal scenarios predicted a population size of approximately 850 individuals under the proactive conservation scenario that redistributes PAs (scenario 2). Scenario 3, proactive conservation maintaining PAs, was the next best scenario for conserving the total population size, with a projected population size of about 625 individuals for both dispersal abilities. Scenario 4, expedient conservation redistributing PAs, was the next most effective, with a simulated population size of about 600 for both dispersal abilities. Scenario 5 (expedient conservation maintaining PAs) was only marginally more effective than scenario 6 (existing PAs effective), resulting in simulated population sizes of 575 and 560 clouded leopards, respectively. These scenarios were also not substantially different from scenario 7, which did not maintain existing protected areas and did not add new ones (predicted population size of 545).

Validation of the forest loss layer presented in Cushman, Macdonald et al (2017)

The risk of forest loss was validated against observed forest loss during the same period by comparing Cushman, et al. [1]to Hansen, et al. [2]. Hansen et al define forest as "canopy closure for all vegetation taller than 5m in height" and forest loss as "a change from a forest to non-forest state". This definition of forest means Hansen may occasionally mis-classify mature plantations as forest. Future work should compare forest loss projections against a range of land cover datasets, but this was beyond the scope of this study.

Hansen, et al. [2] provide data on the year of forest loss between 2000 and 2022. Pixels that were lost between 2010 and 2020 were coded as 1 (loss) and all forested pixels that did not experience loss were coded as 0.

We evaluated several attributes of the observed Hansen, et al. [2] and predicted Cushman, et al. [1] loss. First, we compared the total amount of observed vs. predicted loss. Second, we mapped differences in the amount of observed and predicted loss in local (5km radius) landscape areas to describe spatial patterns and nonstationarity in forest loss across Borneo. Third, we extracted the Hansen et al. (2013) loss vs. no loss (0/1) and the Cushman, et al. [1] predicted loss risk for 1,000,000 randomly chosen pixels that were forested in 2010, and calculated AUC, precent observations correctly classified, sensitivity and specificity (the latter 3 at the maximum Kappa cut-point) using the PresenceAbsence package[3] in R[4].

Supplementary Table 1: Comparison of predicted and observed forest loss.

sum risk	232600.4
sum loss	257670
ratio	1.10778

The sum of the risk surface (probabilities across pixels) equals the expected number of forest loss pixels between 2010 and 2020 Cushman, et al. [1]. The observed number (from the Hansen, et al. [2] loss layer) was 10.8% higher than the expected value. This indicates an acceleration of 10% in the rate of forest loss from 2010 to 2020. In other words, deforestation accelerated from the already very high level in 2000-2010 in the period between 2010-2020.

Supplementary Table 2. Validation of the deforestation risk layer

	threshold	PCC	senitivity	specificyty	kappa	auc
Borneorisk	0.241379	0.71	5.10E-01	0.76	2.30E-01	0.71

The AUC of the deforestation risk layer, tested against actual loss between 2010 and 2020 (from the Hansen loss layer) was 0.71, which is a fairly good prediction given the stochastic process of loss across a risk layer. Assessed at the maximum Kappa cut-point, 71% of the pixels were correctly

classified. The sensitivity was lower than the specificity (0.51 vs 0..76) suggesting that it is easier to predict where deforestation did not occur as compared to where it did occur. This is the same pattern as the original model[1], and shows that where risk is low deforestation will not occur, but where it is relatively high there is more variation as to the stochastic process of which cells are actually lost.

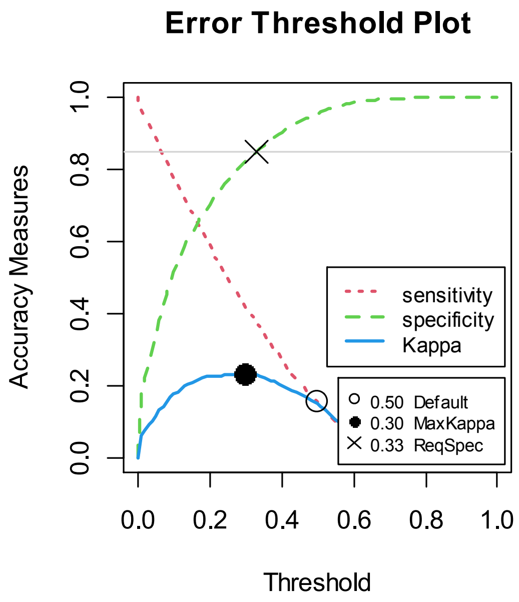

Supplementary Figure 4. AUC Error Threshold Plot

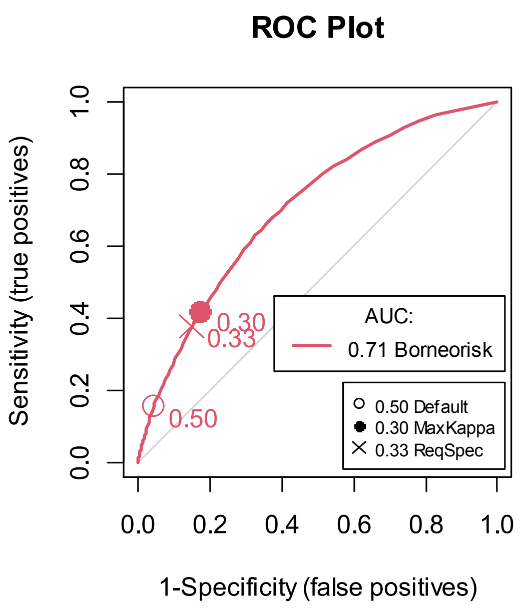

Supplementary Figure 5. AUC ROC plot

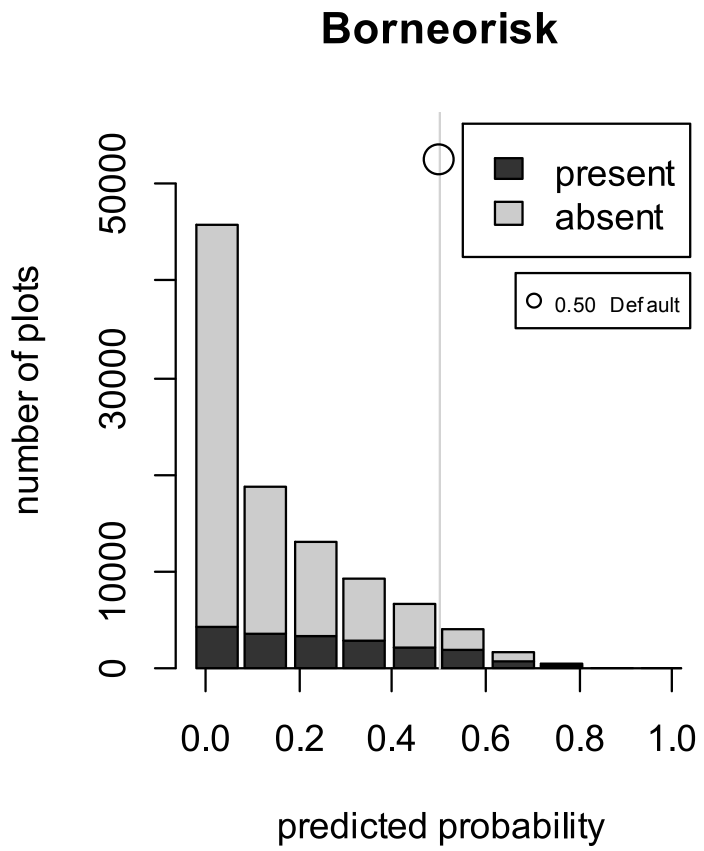

Supplementary Figure 6. Plot of loss vs not across the predicted probability

These show a monotonic and linear relationship in the ratio of loss to not loss across predicted risk. This indicates that the model predicts loss moderately well (as indicated by the 0.71 AUC). However, the relatively large number of actual loss pixels in cells predicted to be relatively low risk suggests that the drivers and patterns of deforestation have changed between 2010-2020 as compared to 2000-2010. A map of the difference in observed vs predicted loss in a 5km radius focal neighbourhood shows where this risk change occurred.

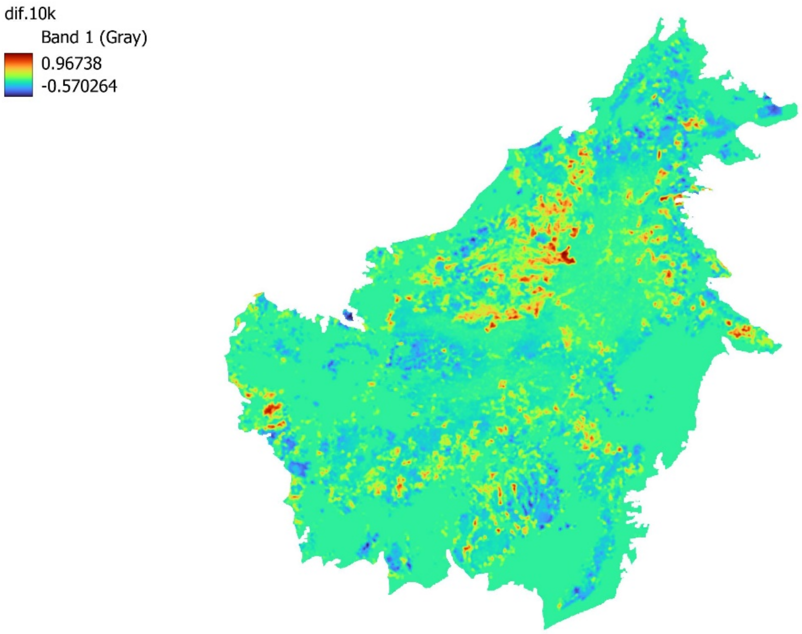

Supplementary Figure 7. Observed – Risk neighbourhood difference map.

In the observed forest loss – risk difference map (Figure S7), red areas are those that had forest loss but that were predicted to have a low risk of loss. Blue areas are areas that did not experience forest loss but had a relatively high predicted risk of loss. The map shows most of the acceleration in forest loss and error in our prediction is related to increased deforestation at higher elevations and rougher landscape topography in E and NE Sarawak. There was also patchy elevated deforestation risk along the forest frontier in NE and S Kalimantan. Forest loss seems to have decreased in Sabah (eg little red and much blue).

Supplementary References

1 Cushman, S. A., Macdonald, E. A., Landguth, E. L., Malhi, Y. & Macdonald, D. W. Multiple-scale prediction of forest loss risk across Borneo. *Landscape Ecology* **32**, 1581-1598 (2017). https://doi.org:10.1007/s10980-017-0520-0

2 Hansen, M. C. *et al.* High-Resolution Global Maps of 21st-Century Forest Cover Change. *Science* **342**, 850-853 (2013). https://doi.org:doi:10.1126/science.1244693

3 Freeman, E. A. & Moisen, G. PresenceAbsence: An R Package for Presence Absence Analysis. *Journal of Statistical Software* **23**, 1 - 31 (2008). https://doi.org:10.18637/jss.v023.i11

4 R: A Language and Environment for Statistical Computing (R Foundation for Statistical Computing, Vienna, Austria, 2021).
